# Natural Compounds That Target Glioma Stem Cells

**DOI:** 10.3390/neurosci6020052

**Published:** 2025-06-05

**Authors:** Mariia Yaroshenko, Monika Christoff, Mateusz Ścibiorski, Karolina Surowiec, Joanna Jakubowicz-Gil, Joanna Sumorek-Wiadro

**Affiliations:** 1 Faculty of Biology and Biotechnology, Maria Curie-Skłodowska University, Akademicka 19, 20-033 Lublin, Poland; yaroshenkomariia14@gmail.com (M.Y.); monikachristoff1@gmail.com (M.C.); karolina.surowiec898@gmail.com (K.S.); 2 Department of Functional Anatomy and Cytobiology, Maria Curie-Sklodowska University, Akademicka 19, 20-033 Lublin, Poland; mateusz.scibiorski@mail.umcs.pl (M.Ś.); joanna.jakubowicz-gil@mail.umcs.pl (J.J.-G.)

**Keywords:** glioma stem cells, glioma treatment, glioblastoma, natural compounds

## Abstract

Gliomas are the most common central nervous system tumors and account for 30% of all primary brain tumors, 80% of all malignant ones, and the vast majority of deaths that are caused by brain tumors. Among them, glioblastoma multiforme has the most aggressive and invasive course. Due to its heterogeneity, it is difficult to treat, and one of the reasons for this are glioma stem cells (GSCs). Therapies such as radiotherapy and chemotherapy are used to treat gliomas but do not bring the expected results. Therefore, treatments targeting glioma stem cells are emerging. A promising strategy is to target GSCs with natural compounds. This review aims to describe the problem of glioma stem cells, the treatment of gliomas, and therapies based on natural compounds, which are promising for the future.

## 1. Introduction

Gliomas are the most common primary malignant brain tumors arising from glioma cells. Every year, roughly 100,000 people worldwide are diagnosed with diffuse gliomas. Discoveries over the past decade have deepened our understanding of the molecular alterations underlying glioma and have identified numerous heritable genetic risk factors [1]. Glioblastoma is known as the most common primary brain malignancy in adults and one of the most aggressive cancers. It accounts for 49% of all primary brain tumors [2]. On histopathological imaging, GBM is characterized by rich cellularity and a high proliferation rate. In addition, palisade-like arrangements of cells occurring around necrotic foci are observed in the microscopic image. Also typical of GBM is the peculiar vascularization and the presence of Scherer structures, i.e., the accumulation of tumor cells near neurons and blood vessels. Such localization of tumor-transformed cells indicates their high migratory and invasive potential [3]. Current treatment protocols include surgical resection combined with radiochemotherapy. Due to the extremely infiltrative nature of gliomas, macroscopic removal of tumor tissue is virtually impossible. Therefore, surgical treatment is aimed at maximal removal of the tumor that will not lead to neurological deficits. Current therapeutic protocols include the administration of temozolomide (TMZ) in combination with targeted radiotherapy. The aim of the simultaneous application of ionizing radiation and a chemotherapeutic agent is to intensify the cytotoxic effect, both locoregionally (destruction of the primary tumor) and spatially (elimination of micro-metastases). The main effect of ionizing radiation on cells is DNA strand breaks and mitotic catastrophe, while TMZ, as an alkylating agent, leads to the formation of O6-methylguanine, resulting in cell cycle inhibition and apoptosis [4,5].

The treatment of gliomas is very difficult, both because of the sheer location of the tumors and their infiltrative nature and because they are highly resistant to the forms of treatment used. One reason for this is the remarkable heterogeneity of the tumor, within which glioma stem cells (GSCs) are found alongside differentiated cells. These have the ability to self-renew and initiate tumor growth and are responsible for the induction of systemic immunosuppression [6].

In recent years, a vast amount of knowledge has been gathered regarding the molecular characteristics of gliomas, especially glioblastoma, e.g., alterations in signaling pathways, cellular microenvironments, and immunogenicity, among others. Thanks to this knowledge and a better understanding of the mechanisms of cell damage and cell death induced by radiotherapy and chemotherapy, it is possible to introduce various therapies and discover new ones. One of the prospects for the future is compounds of natural origin that cause tumor reduction and potentiate existing therapies [7,8].

Therefore, the purpose of this review is to present the current state of knowledge on the use of plant-derived compounds in the elimination of glioma stem cells. This will allow researchers to systematize their existing knowledge and facilitate the planning of future research. The chapter proper, covering the use of natural compounds in the elimination of GSCs, is preceded by a brief characterization of glioma stem cells.

## 2. Glioma Stem Cells

Cancer stem cells (CSCs) are undifferentiated cells capable of transforming into all types of cells that build tumor mass, including tumor angiogenesis cells. Generally, CSCs have characteristics similar to those of normal stem cells but are additionally oncogenic [9]. They are directly associated with high tumor heterogeneity and represents a pool of chemoresistant cells responsible for the progression and recurrence of many types of cancer. Depending on their metabolic activity, CSCs may be in a proliferative or dormant state. Conventional chemotherapy targets highly proliferative cells; however, reducing metabolic activity and going into a dormant state allows CSCs to survive chemotherapy and develop more resistant phenotypes [10,11]. Dormant CSCs can enter a proliferative state under the influence of extracellular factors (such as oxygen availability), activating the cell cycle, oxidative phosphorylation, and key enzymes in the TCA cycle. In contrast, hypoxic conditions promote CSCs in a dormant state with reduced metabolism and oxidative phosphorylation. CSCs also show radioresistance, correlated with increased expression of markers such as CD133 and SOX2, responsible for inducing DNA repair [12]. At the molecular level, this is due to malfunctioning signaling pathways, such as the highly over-regulated Notch, Wnt/β-catenin, and SHH pathways, as well as impaired BMP, NF-κB, and EGF signaling [13].

To be classified as GSCs, cells should possess the following characteristics: self-renewal properties, multipotency (capable of differentiation), and the ability to initiate tumors in animal models [14]. They contribute to disease progression and relapse due to their resistance to current therapies, making glioma difficult to treat. GSCs are a source of high tumor heterogeneity and occupy specific areas of the tumor that are suitable for their growth, proliferation, and survival. They can occupy perivascular, necrotic, and invasive niches. In perivascular niches, GSCs maintain stemness, enhance migration and DNA repair capabilities, and express proteins like MMP9 [15]. They remodel and maintain these niches by secreting proangiogenic factors such as VEGF [16]. Another environment inhabited by GSCs is the necrotic niche. Here, metabolism is reprogrammed to aerobic glycolysis and glutamine-derived fatty acid generation. A key factor involved in this process is HIF-1α, which induces mesenchymal shift, generates survival factors like ERK, and promotes angiogenesis in hypoxic regions [15]. The third GSC microenvironment is the invasive niche, where cells acquire invasive properties responsible for tumor recurrence after surgical resection [17]. These cells are able to spread diffusely using blood vessels as well as white matter tracts using specialized proteins including cadherins, integrins, and metalloproteinases. The invasion process is extremely complex, as it involves multiple signaling pathways and EMT-inducing factors. The three types of microenvironment mentioned are attractors that form the tumor structure, create conditions that promote the formation of multiple subpopulations of GSCs, and contribute to chemo- and radioresistance [15].

**Figure 1:**
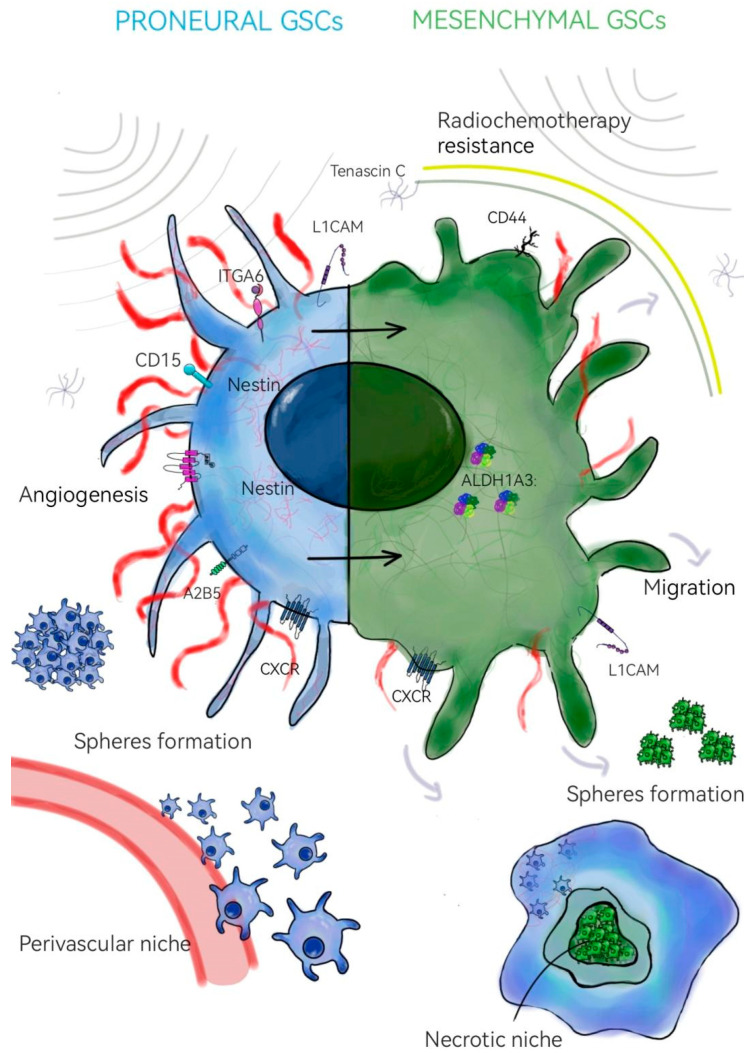
Differences between proneuronal (blue) and mesenchymal (green) glioma stem cells (GSCs).

The existence of different biomarkers, phenotypes, and gene signatures has allowed the division of GSCs into two main subtypes: mesenchymal (MES) and proneuronal (PN) (Figure 1). PN GSCs prefer the perivascular niche, show a high proliferative index, and can promote angiogenesis and, in vitro, have the ability to form larger spheres compared to MES GSCs. Interestingly, primary PN GSCs can recur as MES tumors after radiotherapy or chemotherapy [18]. There are two hypotheses that explain this phenomenon. The first assumes the PN phenotype transitions into MES, while the second assumes the existence of more treatment-resistant MES GSCs in primary PN tumors. The MES GSC subtype is mainly located in the necrotic niche and shows strong invasive capabilities. Increased lipid and glutamine metabolism of this GSC subpopulation determines higher resistance to radiochemotherapy, contributing to tumor recurrence. The aforementioned GSC subtypes are characterized by the presence of characteristic biomarkers. In the case of MES GSCs, these are mainly CD44 and ALDH1A3, while in PN GSCs, these are CD133, CD15, ITGA6, nestin, and A2B5 [19,20].

Prominin 1 (CD133) is one of the best-known GSCs molecular markers. This transmembrane glycoprotein is responsible for maintaining normal cell membrane dynamics. In addition, through binding to cholesterol, it plays an important role in maintaining adequate lipid concentrations in the cell membrane. Moreover, recent studies indicate that CD133 is a key regulator of cell signaling. Through its involvement in the PI3K/Akt, Src-FAK, Wnt/β-catenin, TGF-β/Smad, and MAPK/ERK pathways, it is responsible for cell proliferation, differentiation, and migration or intercellular communication. However, despite the fact that CD133+ cells are more tumorigenic and have better self-renewal properties, CD133- cells also have the ability to generate tumors; therefore, CD133 is not a universal marker for GSCs [21,22].

Another protein specific to glioma stem cells is Sox2. This protein is one of the transcription factors belonging to the Sox family. Originally, its expression was observed in developing embryos, where it played a key role in the formation of pluripotent cells. In the case of cancer stem cells, in addition to maintaining multipotency, Sox2 is also responsible for self-renewal [14]. Moreover, the presence of Sox2 in gliomas is strongly associated with disease recurrence and resistance to temozolomide treatment [23].

In GSCs, the expression of nestin, a protein found in dividing cells during the early stages of CNS development, is observed. Nestin is an intermediate filament protein and participates in the organization of the cytoskeleton. It participates in the basic processes of stem cells: self-renewal, proliferation, differentiation, and migration [24]. In gliomas, high nestin expression correlates with higher tumor malignancy and worse prognosis. In addition, the presence of the protein is also associated with chemoresistance [23].

Another marker of great importance is CXCR4. It is a transmembrane G protein-coupled protein that is a receptor for stromal derived factor 1 (SDF-1). CXCR4 performs, together with its ligand, major functions in mobilizing stem cells into the bloodstream, thereby promoting the metastatic process. In addition, the protein is involved in angiogenesis [25]. Other markers like CD44, CD90, CD184, CD15, A2B5, ALDH1, L1CAM, KLF4, OCT-4, NANOG, SALL4, and GFAP also are used for GSC identification; however, none of them are universal [8].

In addition to increased synthesis of membrane and intracellular proteins, GSCs are also characterized by increased synthesis of tenascin C—an extracellular matrix glycoprotein. Expression of tenascin C is observed in nervous system stem cell niches and plays an important role in neurogenesis. Tenascin has also been shown to be present in the glioma microenvironment and glioma stem cell niches. Its presence in the cerebrospinal fluid is an unfavorable predictor of prognosis and progression of gliomas, promoting processes such as neovascularization, proliferation, adhesion, migration, and immunomodulation [26].

GSCs’ high resistance to chemotherapy is also due to the extraction of the drugs via active transport with ABC-type transporters. They translocate compounds across the cell membrane using energy derived from ATP hydrolysis. In the body, they are present in organs with a high metabolic rate and co-construct the blood–brain barrier, taking part in the elimination of dangerous compounds such as toxins, drugs, or metabolites. Their increased expression, observed in both dedifferentiated and glioma stem cells, prevents the achievement of adequate therapeutic drug concentrations in cancer cells [27].

Another factor facilitating the chemoresistance of GSCs is their cell cycle. While many types of chemotherapy target different checkpoints of the active cell cycle, GSCs mostly remain dormant and therefore are not susceptible to such treatment [14]. The hypoxic microenvironment of GSCs also contributes to chemoresistance. It maintains GSCs in an undifferentiated state by the activation of specific HIF-2α-induced genes [9]. GSCs have better mechanisms for DNA repair, which promotes their resistance to radiotherapy; the notch and TGF-β pathways also contribute to radioresistance. CD133+ cells are capable of avoiding radiation-induced apoptosis by the activation of Chk1 and Chk2 kinases [14].

## 3. Natural Compounds Targeting GSCs

Given the high resistance of glioma stem cells to the forms of treatment used, they can cause rapid relapses. For this reason, compounds are being sought that will target both differentiated and stem cells. Of great importance in this regard are compounds of natural origin. The table below (Table 1) lists the plant-derived compounds that have been studied so far, which may have potential applications in eliminating glioma stem cells and whose detailed mechanisms of action are described later in this article.

Two extremely important features of naturally derived compounds are their much lower toxicity and fewer side effects compared to synthetic drugs. This is important in the case of cancer, where conventional methods are very destructive. Despite the many advantages that natural compounds have, there are some limitations in the standardization or quality control of the research conducted. Studies on natural therapies often differ in methodology, making it difficult to compare the results obtained [62]. In addition, there may be some differences in the composition or purity of the compounds studied, which can also distort the results. Limited bioavailability or, in the case of neurological disorders, the ability to penetrate the blood–brain barrier can also be a problem. In the case of synthetic compounds, the solution may be the attachment of appropriate substituents, while in compounds of natural origin, one alternative may be the use of specific vehicles [63].

Knowledge of the aforementioned parameters allows us to assess the possibility of using natural compounds in medicine. Therefore, the following table summarizes the bioavailability, BBB penetration capacity, and side effects caused by the compounds described later in this article (Table 2).

### 3.1. Phenolic Compounds and Flavonoids

Phenolic compounds are known as metabolites derived from secondary pathways of plants with a typical aromatic ring containing one or more hydroxyl groups. Their representation group is flavonoids, possessing a number of medical benefits, such as anti-inflammatory, antimicrobial, antiatherosclerotic, antidiabetic, antiallergic, prebiotic, and antimutagenic effects. Also, phenolic compounds are essential components of food. In addition to their strong antioxidant properties, they influence the sensory characteristics of food products [102]. Moreover, phenolic compounds and flavonoids exhibit anticarcinogenic activity by influencing molecular mechanisms such as the Wnt/β-catenin signaling pathway, PI3K/AKT/mTOR, NF-κB, or Ras/Raf/MEK/ERK. These compounds may be of great interest in the near future in the context of research on glioma stem cells [103,104].

#### 3.1.1. Resveratrol

Resveratrol (RSV) is a polyphenolic phytoalexin occurring naturally in fruits, berries, and vegetables. The compound is produced by plants in response to environmental stress, enabling them to survive in adverse conditions. It acts as a phytoestrogen and is well known for its antioxidant and anti-inflammatory effects. Moreover, RSV exhibits a broad spectrum of anticancer activity through its antiproliferative, proapoptotic, and antimigratory activities. One of the advantages of this compound is its ability to cross the blood–brain barrier [105].

Recent studies by Cilibrasi et al. have shown that RSV also has anticancer activity against primary GBM-derived GSCs (GBM2, GBM7, G144, G179, G166, GliNS2, and GBM04). The compound significantly reduces the metabolic rate of GSCs, as well as their mitotic activity, which is crucial for cancer proliferation. Moreover, RSV also reduces the activity of the Wnt/β-catenin signaling pathway, the overexpression of which is observed in many types of cancer [28]. It is responsible for cell growth, proliferation, and tissue development. Transmembrane receptors associated with the Wnt pathway are Frizzled receptors and low-density lipoprotein receptors (LRP5/6). Both receptors, together with Wnt (Wnt proteins, such as Wnt3a, Wnt1, and Wnt5a), form a trimeric complex activating signal transduction. The primary consequence of Wnt pathway activation is the blocking of the activity of the complex marking β-catenin for degradation. Therefore, β-catenin is able to translocate to the nucleus, where it interacts with TCF/LEF and activates the transcription of genes responsible for cell proliferation, survival, differentiation, and migration [106]. In the absence of the stimulation of these receptors, β-catenin is degraded, thereby preventing the transcription of target genes. The study showed that RVS completely eliminates β-catenin in sensitive GSC cell lines. Moreover, the level of c-Myc protein, controlled by one of the target genes of the Wnt pathway, reduces. This is likely due to the activation of post-translational mechanisms, for example, by Sirt1, which destabilizes c-Myc stability [28].

In addition, lower cell motility was observed in all the GSCs studied, resulting in reduced infiltration—one of the main features of GSCs. RSV is able to inhibit epithelial–mesenchymal transition (EMT) by decreasing the activity of two key transcription factors, Twist1 and Snail1. EMT is a process of cell transformation from the epithelial type with cell-to-cell adhesion properties and polarity into mesenchymal-like cells, which are more invasive and motile [28].

#### 3.1.2. Luteolin

Luteolin is a common flavonoid that exists in many types of plants, including fruits, and vegetables such as celery, chrysanthemum flowers, sweet bell peppers, carrots, broccoli, parsley, and medical herbs [107]. Plants rich in luteolin have been used in Chinese medicine for treating various diseases such as hypertension, inflammatory disorders, and cancer. Luteolin’s anticancer properties are associated with the induction of apoptosis and inhibition of cell proliferation, metastasis, and angiogenesis [108].

Studies on primary GBM-derived GSCs have shown that the therapeutic effect of luteolin is dose-dependent, with optimal concentrations (50 μM) reducing GSC survival by about 60% compared to controls. At the same time, it was observed that the compound can increase the cytotoxicity of temozolomide by suppressing cell survival pathways such as PI3K/AKT/mTOR, NF-κB, or Ras/Raf/MEK/ERK. The PI3K/AKT pathway is the most common activated pathway in cancers. It is activated downstream of receptor tyrosine kinases (RTKs), cytokine receptors, integrins, and G-protein-coupled receptors (GPCRs) and plays a central role in promoting GSCs’ survival and growth. This pathway regulates key metabolic processes, including glucose metabolism, the biosynthesis of macromolecules, and the maintenance of redox balance to support systemic metabolic homeostasis and the growth and metabolism of individual cells. In cancer cells, this pathway reprograms cellular metabolism by augmenting the activity of nutrient transporters and metabolic enzymes, thereby supporting the anabolic demands of aberrantly growing cells [29].

Another mechanism of luteolin’s anticancer activity is the compound’s ability to induce apoptosis. At the molecular level, this is associated with increased expression of caspase 3 and 7, which are known to be effector caspases during the cell death cascade. [29,108]. Luteolin succeeded in the upregulation of proapoptotic mediators as caspases and the Bcl-2 family and cell cycle controllers as p53 and p27 [30].

It has also been shown that luteolin can reduce the autophagy process in GSCs [30]. In light of recent reports of the possibility of using this process as a mechanism of survival of cancer cells, the results obtained appear to be highly desirable. It has been observed that inhibition of the autophagy process significantly increases the effectiveness of the anticancer therapies used. Autophagy may also be responsible for developing resistance to the chemotherapeutics used by protecting cancer cells from programmed death [109,110].

#### 3.1.3. Curcumin

Curcumin is a polyphenolic compound occurring naturally in the turmeric rhizomes (*Curcuma longa*). It is often used in traditional medicine, due to the number of its therapeutic properties, such as anti-inflammatory, antioxidative, hepatoprotective, analgesic, antiseptic, and antitumor effects [111].

Curcumin has also been shown to have anticancer properties against glioma stem cells. Gersey et al. described that curcumin inhibits the sphere-forming ability and colony-forming potential of primary GBM-derived GSCs (Glio3, Glio4, Glio9, Glio11, and Glio14) [31].

Curcumin is capable of reducing GSC viability and proliferation rates and also induces apoptosis via activating the MAPK pathway and reduces the activity of STAT3 and IAP proteins [31]. The MAPK signaling pathway has multiple roles in the cell signaling process and can lead to different outcomes depending on the cascade activated. There are four types of MAPK cascades: ERK, JNK, p38 MAPK, and ERK5. MAPK/ERK activation causes subsequent reactions of a cascade of enzymes: Ras, Raf, MEK, and ERK. This activates target genes responsible for the proliferation of cells, differentiation, apoptosis suppression, angiogenesis, and tumor metastasis. On the other hand, JNK and p38 MAPK are associated with stress response and apoptosis induction [112]. The IAP protein family comprises inhibitor proteins of apoptosis. They deactivate caspases and influence ubiquitin (Ub)-dependent signaling pathways that activate the MAPK pathway and NF-κB production, responsible for cell survival [113]. STAT3 is a part of the JAK/STAT signaling pathway. It is responsible for cell growth regulation, immunosuppression, and apoptosis inhibition, as well as enhances cell resistance to reactive oxygen species (ROS)-induced apoptosis [114]. Therefore, a decrease in the activity of the mentioned protein sensitizes GCSs to the action of ROS generated as a result of the action of curcumin. ROS are natural byproducts of oxygen metabolism. In regulated states, they play a role in cell signaling and maintenance of homeostasis. But when imbalanced, ROS cause oxidative stress and, subsequently, cell proliferation and apoptosis [31].

#### 3.1.4. Apigenin

Apigenin is a flavonoid of natural origin that is very widely distributed, as it is found in many common edible plants and healthy foods [115]. To date, research using this naturally derived compound concentrates on the radioresistance of GSCs and the effect on HIF-1α, an important regulator of the glucose metabolic pathway in cancer cells [32]. Studies have shown that, under the influence of apigenin, there is a reduction in the expression of HIF-1α and the associated GLUT-1/3, NF-κB, p65, and PKM2 proteins, whose increase is induced by radiation. These results suggest that the sensitization of GSCs to radiation may be linked to the levels of HIF-1α and its associated aforementioned proteins [33].

### 3.2. Alkaloids

Alkaloids are natural bioactive compounds containing nitrogen. They can be found primarily in plants, fungi, and occasionally animals. Compounds that belong to this group have complex and varied structures, which contribute to their biological effects. They are characterized by antimicrobial, anti-inflammatory, neuroprotective, and analgesic effects and the promotion of cardiovascular health effects. In anticancer therapies, alkaloids can be used as they have the ability to inhibit cell proliferation and induce apoptosis. The mechanisms are different for different representatives of the group, but one of the most common is the inhibition of microtubule polymerization and mitotic arrest, activation of caspase-dependent apoptosis via the mitochondrial pathway, inhibition of angiogenesis through reductions in vascular endothelial growth factor (VEGF) signaling, DNA topoisomerase inhibition, induction of oxidative stress-mediated apoptosis, and other interferences crucial for cancer cell signaling pathways [116,117].

#### 3.2.1. Harmine

Harmine is a naturally occurring β-carboline alkaloid isolated from *Peganum harmala*. Harmine has many pharmacological activities, including anti-inflammatory, neuroprotective, antidiabetic, and antitumor activities. What is more, harmine exhibits insecticidal, antiviral, and antibacterial activities [34]. Recent studies have shown that harmine can inhibit cancer cell proliferation and metastasis through epithelial-to-mesenchymal transition, cell cycle regulation, and induction of tumor cell apoptosis [35].

Harmine inhibits self-renewal and induces GCS differentiation, in particular, inhibiting neurosphere formation of primary GBM-derived GSCs (GBM-3) and GSCs isolated from cell lines (C6, U87), which is associated with a decrease in CD133 expression. Moreover, harmine inhibits the EGF-mediated phosphorylation of AKT. AKT protein in the unphosphorylated form is inactive, resulting in the inhibition of intracellular signaling promoting proliferation, angiogenesis, and migration [8,36,37]. Another mechanism of harmine activity against GSCs is based on the compound’s ability to induce apoptosis by increasing the active form of caspase 3 [37].

#### 3.2.2. Cyclopamine

Cyclopamine is a steroidal jerveratrum alkaloid found in plants of the *Veratrum genus*. This chemical compound is characterized by a unique skeletal structure (Table 1). Studies conducted to date have shown that cyclopamine can be used in the treatment of psoriasis and has shown anticancer properties against many types of cancer (including head, neck, and ovarian cancers). The mechanism of this activity is based on the compound’s ability to inhibit the Sonic Hedgehog (SHH) pathway. During the embryonic period, this pathway is highly involved in the regulation of migration and specification of many cell populations. After the embryonic period, the activity of the SHH pathway is silenced, and increased activity is observed in tumors. It is responsible for the promotion of tumor growth, as well as the onset of multidrug resistance. The Shh cascade is initiated by the attachment of the Shh peptide to the Patched receptor (Ptch). Ptch in its non-Shh-bound state inhibits the Smoothened (Smo) protein. The attachment of Shh releases Smo from the inhibitory influence of Ptch, and then, active Smo stimulates the transport of Gli proteins into the cell nucleus, where they bind to DNA and trigger the transcription of target genes, such as HOX, WNT, FGF-4, VEGF, CAPN1, and NRP [118,119,120,121].

The anticancer effect of cyclopamine has also been described against GSCs. The compound, by inhibiting the SHH pathway, reduces the survival of stem cells derived from primary tumor cultures. In addition, it reduces the ability to form spheres, which is associated with a decrease in nestin levels. Cyclopamine has also been shown to sensitize GSCs to temozolomide and reduce their invasiveness [38,39,40].

### 3.3. Terpenoids

Terpenoids are secondary metabolites of plants, fungi, and occasionally marine organisms that consist of isoprene units (C5). They are classified into monoterpenes (two isoprene units), sesquiterpenes (three), diterpenes (four), triterpenes (six), tetraterpenes (eight), and polyterpenes (multiple). They have various biological effects, such as anti-inflammatory, antioxidant, antiallergic, antimicrobial, antifungal, neuroprotective, and cardioprotective effects. Terpenoids also exhibit anticancer properties as they inhibit angiogenesis and metastasis and induce apoptosis. They act in various ways, including ROS induction, inhibition of the NF-κB pathway, increases in pro-apoptotic proteins, reductions in VEGF expression, and promotion of autophagy [122,123].

#### 3.3.1. Honokiol

Honokiol has been described as a component of *Magnolia obovata*, which is a component of Asian herbal teas [124]. Previous studies have shown many biological properties of honokiol, including anti-inflammatory, antithrombotic, antiangiogenic, and antitumor effects [41].

Recent studies have shown that honokiol also has antitumor properties against GSCs isolated from the U87 cell line. By decreasing STAT3 phosphorylation, the compound decreases the expression of CD133 and nestin and thus inhibits the formation of GSC spheroids [42].

Another mechanism of action of honokiol on GSCs is the induction of apoptosis by inhibiting the Notch pathway, which also regulates cell differentiation, angiogenesis, and proliferation. The same study showed that honokiol increases the susceptibility of glioma stem cells to temozolomide therapy. Interestingly, the compound also proved to be an effective TMZ adjuvant in the induction of apoptosis of GSCs (primary GBM-derived GSCs—GBM-8401) [43].

#### 3.3.2. Tanshinone IIA

Tanshinone IIA is a compound existing in the rhizome of *Salva miltiorrhiza* Bunge, also called Danshen. Tanshinone IIA exerts pharmacological activity on nervous system disease, endocrine system disease, cardiovascular and cerebrovascular disease, as well as cancer [44,45].

It was shown that tanshinone IIA possesses anticancer, anti-inflammatory, and anti-oxidative activities in vitro and in vivo. Interestingly, the studies showed that GSCs are more sensitive to tanshinone IIA in vitro than normal cells, which suggests that tanshinone IIA possesses stronger inhibitory activity against GSCs. The compound inhibits the proliferation and sphere-forming ability of GSCs isolated from the WJ1 line and inhibits the growth of human brain tumors initiated by GSCs in vivo. At the same time, it was shown to be associated with a decrease in the expression levels of GSC marker proteins, including CD133 and nestin. One of the important roles in the maintenance and progression of GSCs is played by inflammatory cytokines. In the GSC expression levels of IL6, phospho-STAT3 (tyrosine705) and phospo-STAT3 (serine727) are high. After treatment with tanshinone IIA, IL6, phospo-STAT3 (tyrosine705), and phospho-STAT3 (serine727) were decreased, which suggested that tanshinone IIA has the potential to block inflammatory signaling pathways in GSCs by downregulating pathway-related protein expression [46].

Tanshinone IIA, in addition to its ability to reduce GSC stemness, also has proapoptotic activity. At the molecular level, this is related to the compound’s ability to increase the levels of Bax protein and cleaved caspase 3 and decrease the anti-apoptotic Bcl-2 protein [46].

#### 3.3.3. Cannabinoids

Cannabinoids are bioactive substances of the plant *Cannabis sativa*. They have an extremely wide range of pharmacological properties, and they can be used to treat neurological disorders such as epilepsy, multiple sclerosis, insomnia, spasticity, vomiting, nausea and pain, and cancer [125]. Generally, they act as agonists of G-protein-coupled cannabinoid receptors [47].

In GSCs (derived from glioblastoma multiforme biopsies and U87 and U373 cell lines), cannabinoids bind to cannabinoid receptors (CB1 and CB2), initiating cell differentiation and inhibiting gliomagenesis. It is correlated with decreased expression of the characteristic marker nestin, which indicates a loss of stemness. Moreover, cannabinoids also reduce the ability of GSCs to initiate glioma formation in vivo, which is associated with reduced neurosphere formation and decreased proliferation [48].

Another mechanism of cannabinoids is based on their ability to inhibit viability and induce apoptosis in primary GBM-derived GSCs (GSC 3832, GSC 387, CPMC146). This activity is associated with an increase in ROS, which in turn increases caspase 3 levels and inhibits p-AKT (phosphorylated AKT). The study also shows the effect of CBD on the self-renewal properties of GSCs, as it is able to reduce Sox2, Id1, and phosphorylated STAT3 markers and activate the p38 MAPK pathway [49].

### 3.4. Other Natural Compounds

#### 3.4.1. Guggulsterone

Guggulsterone is a plant steroid hormone derived from a resin of *Commiphora mukul*, which is a flowering shrub local to some regions in India [50]. Due to its broad biological activity, including anti-inflammatory, antiatherosclerotic, antioxidant, and antihyperglycemic effects, it can be used in the treatment of many diseases. Recent studies have also shown promising anticancer effects, also in relation to glioma stem cells [126]. Guggulsterone reduced sphere formation in patient-derived glioblastoma stem-like cells (CD133+ cells). This ability to reduce GSC proliferation was associated with a decrease in Gli-1 levels, which is a highly upregulated transcriptional factor in GSCs responsible for cell survival and growth. Guggulsterone also reduced GSC stemness by decreasing the levels of nestin and IQGAP-1, markers of glioma stem cells. IQGAP1 is responsible for cytoskeleton organization, signal transduction, and cell adhesion; however, in GSCs, it contributes to their proliferation and invasiveness [50,51]

#### 3.4.2. Sulforaphane

Sulforaphane is a compound within the isothiocyanate group of organosulfur compounds. Isothiocyanates are hydrolysis products of glucosinolates, secondary plant metabolites found in Brassica vegetables [52]. It has been shown that sulforaphane might protect against various types of cancer, may decrease the risk of cardiovascular disease, and help in osteoporosis [53].

Sulforaphane has several anticancer properties, including in glioma stem cells. Bijangi-Wishehsaraei et al. showed that the compound induces apoptosis in CD-133-positive GSCs isolated from the regular cultures of U87MG, SF767, U118, and U373 cell lines. The compound also inhibited sphere formation by GSCs isolated from the U87 and MHBT161 cell lines, as well as from primary cultures [54].

#### 3.4.3. Eckol

Eckol is a precursor compound representing the dibenzo-1,4-dioxin class of phlorotannins, which are abundant in *Ecklonia species*, which are brown marine algae. It has anti-inflammatory, antibacterial, antiviral, neuroprotective, antihistaminic, anticancer, and antiproliferative properties.

Recent studies have shown that eckol can suppress the expression of glioma stem cell markers and self-renewal-related proteins [55]. Hyun et al. showed that the compound reduced the number and size of spheres formed by patient-derived glioma stem cells and GSCs derived from U87MG and U373MG cell lines. At the same time, eckol was observed to reduce the expression of glioma stem cell markers such as CD133, nestin and Musashi-1, Sox2, Notch2, and β-catenin. The decrease in the levels of these proteins led to the loss of self-renewal capacity and reduced tumorigenicity. In addition, eckol has been shown to inhibit the activity of PI3K and Akt proteins and Ras, Raf, and Erk proteins, which are part of the signaling pathways PI3K/Akt/mTOR and Raf/MEK/ERK, respectively. Their activity is crucial for maintaining the properties of initiating cells, so a decrease in the activity of the aforementioned signaling pathways leads to a decrease in the stemness of GSCs [56].

In addition, treatment with eckol reduced the resistance of glioma stem cells to temozolomide and radiotherapy, indicating that the compound may have future applications in increasing the sensitivity of GSCs to anticancer treatments [56].

#### 3.4.4. All-Trans-Retinoic Acid (Vit A Acid)

All-trans-retinoic acid (ATRA) is a metabolite of vitamin A. Vitamin A can be obtained from the diet in the form of provitamin A carotenoids from red and orange fruits and vegetables, as well as green vegetables, for instance, carrots, spinach, and tomatoes. It plays an important role in the biological processes of growth and proliferation, including embryo development [127,128]. Other useful properties of ATRA include gene regulation by binding to nuclear receptors that attach to retinoic acid response elements (RAREs) on DNA and immunoregulation of the tumor microenvironment by upregulating the antitumor activity of CD8+ T cells [129].

Recent studies have shown that ATRA also possesses antineoplastic properties against GSCs isolated from the U87 cell line. The compound reduces invasion and proliferation rates in a dose-dependent manner. Moreover, ATRA possesses antiangiogenic properties by reducing the expression of vascular endothelial growth factor (VEGF) [57]. VEGF is a cytokine family responsible for the formation of blood and lymph vessels. After binding to the receptor, it initiates cell proliferation, migration, and new blood vessel formation. It plays an important role in normal development, tissue repair, and reproduction; however, in cancers, it promotes blood supply to the tumor, therefore facilitating tumor growth and metastasis [58].

ATRA inhibits symmetric division of GCS cells, while inducing asymmetric division. Symmetric division is characteristic of the later stages of tumorigenesis, while asymmetric division is closely related to the stemness of cells, and its loss can lead to tumor growth. Thus, the inhibition of symmetric division by ATRA may indicate the inhibition of tumorigenesis in GSCs [59].

#### 3.4.5. PBI-05204

PBI-05204 is a natural extract isolated from Nerium oleander and mainly contains oleandrin. This compound exhibits anticancer properties in the context of gliomas. It is effective in inhibiting the growth of GBM cells by promoting apoptosis and inhibiting the PI3K/mTOR pathway and the maturation of cells of this tumor type [130]. The extract effectively downregulates key markers for GSCs, i.e., GRP78, CD44, and SOX2, thereby affecting the stemness, proliferation, invasiveness, and resistance of GSCs to radiochemotherapy [60]. PBI-05204 effectively eliminates GSCs by necroptosis using the RIPK1-RIPK3-MLKL signaling pathway and multiple changes in cell ultrastructure involving the ER, mitochondria, and cell membrane. In addition, the action of PBI-05204 decreases sphere formation and the cell viability of GSCs by directly affecting the self-regenerative properties of these cells. Studies to date have shown that this compound can enhance the effects of radiotherapy and chemotherapeutic agents used (e.g., TMZ) and effectively penetrates the blood–brain barrier, which is extremely important in the discovery of new compounds with therapeutic potential for the treatment of diseases of the nervous system [60].

#### 3.4.6. RGWE

RGWE is a natural plant-derived aqueous extract isolated from Ruta graveolens [61]. It has a very rich composition as it contains products, i.e., alkaloids, coumarins, essential oils, or flavonoids [131]. The extract has many beneficial properties, which include antioxidant, anti-inflammatory, and anticancer properties [132]. It exhibits cytotoxicity towards glioma cells, while causing no damage to normal cells. Studies have shown that RGWE reduces the viability of GSCs isolated from patients. However, this effect is not comparable to the results of studies on cell lines, as GSCs are characterized by their capacity for self-renewal and the formation of a diverse pool of tumor cells with a high capacity for DNA repair and cellular drug-removal mechanisms, which is mainly responsible for their resistance to currently used therapeutic strategies [61].

## 4. Conclusions

**Figure 2:**
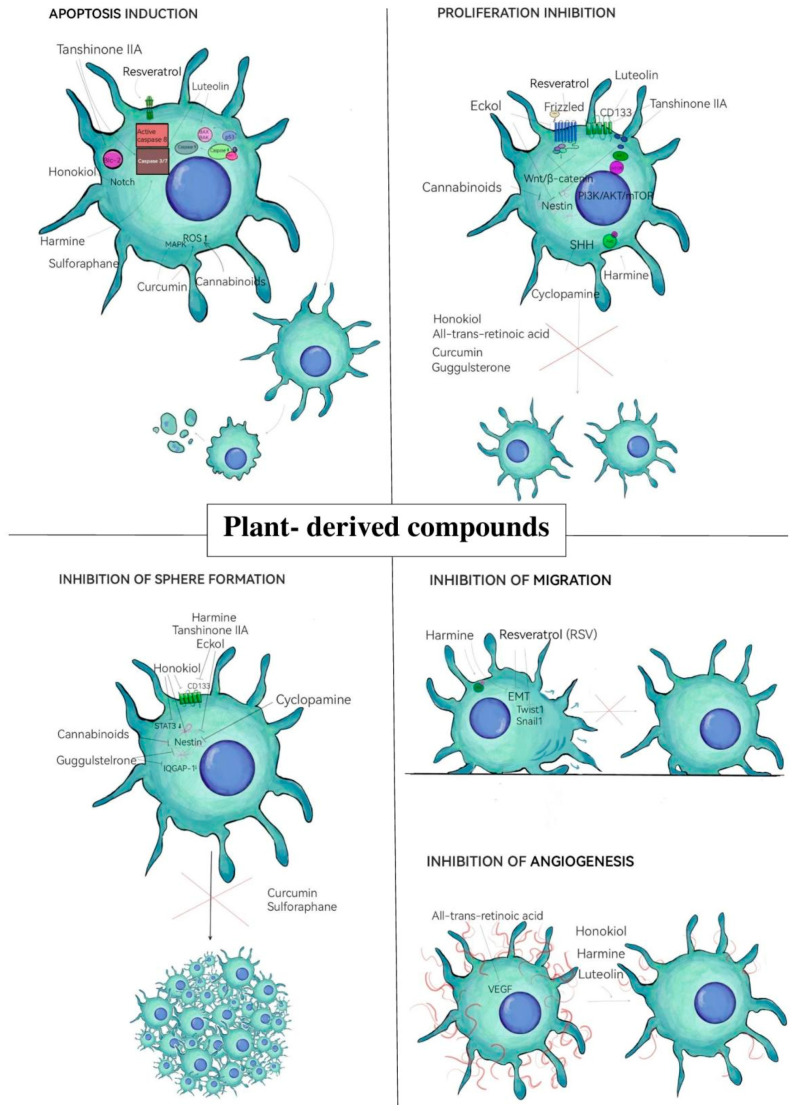
Mechanism of anticancer activity of naturally derived compounds against GSCs.

Gliomas, despite the implementation of intensive treatment in the form of surgical resection and the use of adjuvant radiotherapy in combination with chemotherapy, are characterized by the ability to cause rapid tumor recurrence. One of the reasons for this is the presence of glioma stem cell populations that are resistant to conventional radio- and chemotherapy. This is why it is so important in glioma therapy to use compounds that fight not only differentiated cancer cells but also the cancer stem cell population. In this regard, natural compounds hold great promise as they can be used as adjuvants to currently used therapies. Their additional advantage is their protective effect against normal cells. Previous studies indicate that plant-derived compounds exhibit a range of anticancer properties against GSCs (Figure 2). They possess both proapoptotic and antiproliferative activity, as well as antimigratory activity. In addition, they can affect the stemness of GSCs, preventing sphere formation and inhibiting tumor invasiveness. Given the great potential of plant-derived compounds in eliminating GSCs and the scarcity of literature data on the subject, further research is needed.

## Figures and Tables

**Table 1:** Plant-derived compounds and their effects on GSCs.

Compound		Chemical Structure	Type of GSCs	Mechanisms of Action	Literature
**Phenolic compounds and flavonoids**	Resveratrol	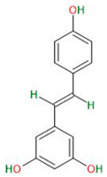	Primary GBM-derived GSCs	Inhibition of proliferation Inhibition of migration	[28]
	Luteolin	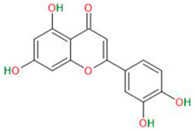	Primary GBM-derived GSCs	Decrease in survival rate Increase in cytotoxicity of temozolomide Induction of apoptosis Inhibition of autophagy	[29,30]
	Curcumin	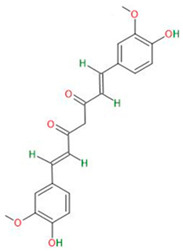	Primary GBM-derived GSCs	Inhibition of sphere formation Reduction in proliferation Induction of apoptosis	[31]
	Apigenin	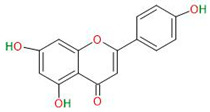	Primary GBM-derived GSCs	Increase in radiosensitivity	[32,33]
**Alkaloids**	Harmine	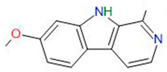	Primary GBM-derived GSCs	Inhibition of proliferation Induction of apoptosis Inhibition of self-renewal	[34,35,36,37]
	Cyclopamine	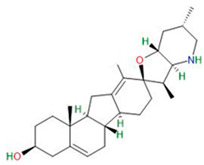	Primary GBM-derived GSCs	Induction of GSC differentiation Reduction in stemness markers Increase in cytotoxicity of temozolomide	[38,39,40]
**Terpenoids**	Honokiol	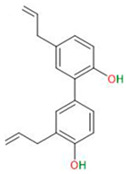	Primary GBM-derived GSCs	Inhibition of sphere formation Induction of apoptosis Increase in cytotoxicity of temozolomide	[41,42,43]
	Tanshinone IIa	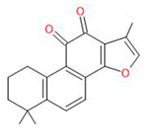	Primary GBM-derived GSCs	Inhibition of proliferation Inhibition of sphere formation Induction of apoptosis	[44,45,46]
	Cannabinoids	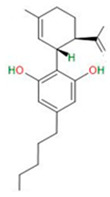	Primary GBM-derived GSCs	Inhibition of sphere formation Induction of apoptosis Initiation of cell differentiation and inhibition of gliomagenesis	[47,48,49]
**Other natural compounds**	Guggulsterone	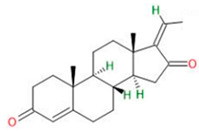	Primary GBM-derived GSCs	Inhibition of sphere formation	[50,51]
	Sulforaphane	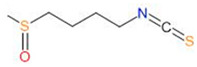	Primary GBM-derived GSCs GSCs isolated from U87, MHBT161, SF767, U118, and U373	Induction apoptosis Inhibition of sphere formation	[52,53,54]
	Eckol	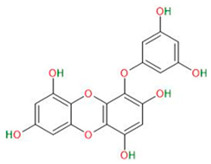	Primary GBM-derived GSCs GSCs isolated from U87 and U373	Inhibition of self-renewal Inhibition of sphere formation	[55,56]
	All-trans retinoic acid (ATRA)	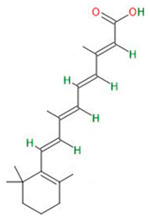	GSCs isolated from the U87	Inhibition of proliferation Inhibition of symmetric cell division and induction of asymmetric cell division	[57,58,59]
	PBI-05204	mixture of compoundsisolated from *Nerium oleander*	Primary GBM-derived GSCs	Inhibition of sphere formation Induction of necroptosis and apoptosis Inhibition of proliferation Reduction in stemness markers Increase in radiochemosensitivity	[60]
	RGWE	mixture of compoundsisolated from *Ruta graveolens*	Primary GBM-derived GSCs	Reduction in viability	[61]

**Table 2:** Oral bioavailability, BBB permeability status, and results of clinical trials of described natural compounds.

Compound		Oral Bioavailability	BBB Permeability	Side Effects in Clinical Trials
**Phenolic compounds and flavonoids**	Resveratrol	77–80% [64]	+ [65]	Well tolerated; relatively few gastrointestinal side effects have been reported (diarrhea, constipation, nausea, abdominal cramps, vomiting, fatty diarrhea, heartburn, reflux, and bloating) [66].
	Luteolin	17.5–53.9% [67]	+ [68]	No significant side effects have been reported [67].
	Curcumin	<1% [69]	- [70]	Mild gastrointestinal symptoms (diarrhea, abdominal pain, flatulence, indigestion, nausea, vomiting, and constipation), headache and dizziness, hair loss, and fever. Individual cases of muscle atrophy and kidney damage [71].
	Apigenin	30% [72]	+ [73]	Well tolerated; no side effects have been reported [73].
**Alkaloids**	Harmine	5–10% [74]	+ [75]	Well tolerated; can cause drowsiness, impaired concentration, and dizziness, but not very often. At higher doses, mild nausea and vomiting occurred [74].
	Cyclopamine	80% [76]	+ [77]	Due to strong side effects in mouse models (weight loss, dehydration, and death), the compound has not been used in clinical trials [77].
**Terpenoids**	Honokiol	5% [78]	+ [78]	Well tolerated; relatively few side effects have been reported (heartburn, trembling hands, sexual dysfunction, thyroid failure, fatigue, and headaches) [79].
	Tanshinone IIa	<4% [80]	+ [81]	No significant side effects have been reported [82].
	Cannabinoids	6–19% [83]	+ [84]	Compounds used in medicine. Can be addictive and intoxicating (depending on the type—THC or CBD) and can cause psychiatric, gastrointestinal, and cardiovascular problems [85,86].
**Other natural compounds**	Guggulsterone	43% [87]	+ [88]	Well tolerated; mild side effects causing dermatologic hypersensitivity have been reported [89].
	Sulforaphane	82% [90]	+ [91]	Well tolerated; mild side effects (constipation and diarrhea) have been reported [92].
	Eckol	<1% [93]	- [94]	Clinical trials have not yet been conducted, and no data on side effects in mice are available [95].
	All-trans retinoic acid (ATRA)	<50% [96]	+ [97]	Well tolerated; mild side effects (fatigue, headache, fever, dermatitis, weakness, and gastrointestinal symptoms) have been reported [98].
	PBI-05204	Different for each component of the extract; about 63% [99]	+ [60]	Well tolerated; relatively few side effects have been reported (vomiting, nausea, decreased appetite, and diarrhea) [100].
	RGWE	Not specified; different for each component of the extract	Not specified, different for each component of the extract	Clinical trials have not yet been conducted, and no data on side effects in rats are available [101].

## Data Availability

Data sharing is not applicable to this article as no new data were created or analyzed in this study.

## Abbreviations

AKT	Protein Kinase B
ALDH1A3	Aldehyde Dehydrogenase 1 Family Member A3
ATP	Adenosine Triphosphate
ATRA	All-Trans-Retinoic Acid
Bax	BCL2-Associated X Protein
Bcl-2	B-Cell Lymphoma 2
BMPs	Bone Morphogenetic Proteins
CAPN1	Calcium-Activated Neutral Protease 1
CB1/2	Cannabinoid Receptor Type 1/2
CD133	Cluster of Differentiation 133
CD15	Cluster of Differentiation 15
CD44	Cluster of Differentiation 44
Chk1 and 2	Checkpoint Kinase 1 and 2
CK1	Casein Kinase 1
c-Myc	Cellular Myelocytomatosis Oncogene
CSCs	Cancer Stem Cells
CXCR4	C-X-C Motif Chemokine Receptor 4
EGF	Epidermal Growth Factor
EGFR	Epidermal Growth Factor Receptor
EMT	Epithelial–Mesenchymal Transition
ERK	Extracellular Signal-Regulated Kinase
FGF-4	Fibroblast Growth Factor 4
GBM	Glioblastoma
GFAP	Glial Fibrillary Acidic Protein
GLUT	Glucose Transporter
GPCRs	G-Protein-Coupled Receptors
GRP78	78 kDa Glucose-Regulated Protein
GSCs	Glioma Stem Cells
HIF-1/2-α	Hypoxia-Inducible Factor 1/2 Alpha
HOX	Homeobox Gene
IAP	Inhibitor of Apoptosis Protein
IL6	Interleukin 6
IQGAP1	IQ Motif Containing GTPase-Activating Protein 1
ITGA6	Integrin Subunit Alpha 6
JAK	Janus Kinase
JNK	c-Jun N-Terminal Kinase
KLF4	Kruppel-Like Factor 4
L1CAM	L1 Cell Adhesion Molecule
LEF	Lymphoid Enhancer-Binding Factor
MAPK	Mitogen-Activated Protein Kinase
MEK	Mitogen-Activated Protein Kinase
MES	Mesenchymal
MGMT	O6-Methylguanine-DNA Methyltransferase
MMP9	Matrix Metalloproteinase 9
MnSOD	Manganese Superoxide Dismutase
mTOR	Mammalian Target of Rapamycin
Musashi-1	Musashi RNA-Binding Protein 1
NANOG	Homeobox Transcription Factor NANOG
NF-κB	Nuclear Factor Kappa-Light-Chain-Enhancer of Activated B Cell
NRP	Neuropilin
O6MeG	O6-Methylguanine
OCT-4	Octamer-Binding Transcription Factor 4
PI3K/Akt	Phosphatidylinositol 3-Kinase/Protein Kinase B
PKM2	Pyruvate Kinase M2
PN	Proneuronal/Proneural
Ptch	Patched Receptor
PTK	Protein Tyrosine Kinase
Raf	Rapidly Accelerated Fibrosarcoma
RAREs	Retinoic Acid Response Elements
Ras	Rat Sarcoma
ROS	Reactive Oxygen Species
RSV	Resveratrol
RT	Radiotherapy
RTKs	Receptor Tyrosine Kinases
SALL4	Sal-Like Protein 4
SHH	Sonic Hedgehog Pathway
SMO	Smoothened Protein
Sox2	SRY-Box Transcription Factor 2
Src-FAK	Src Family Kinases–Focal Adhesion Kinase
STAT	Signal Transducer and Activator of Transcription
TCA	Tricarboxylic Acid Cycle (Krebs Cycle)
TCF	T-Cell Factor
TGF	Transforming Growth Factor
TMZ	Temozolomide
Ub	Ubiquitin
VEGF	Vascular Endothelial Growth Factor
WHO	World Health Organization
